# Protocol: A Multiplexed Reporter Assay to Study Effects of Chromatin Context on DNA Double-Strand Break Repair

**DOI:** 10.3389/fgene.2021.785947

**Published:** 2022-01-31

**Authors:** Ruben Schep, Christ Leemans, Eva K. Brinkman, Tom van Schaik, Bas van Steensel

**Affiliations:** ^1^ Oncode Institute and Division of Gene Regulation, Netherlands Cancer Institute, Amsterdam, Netherlands; ^2^ Department of Cell Biology, Erasmus University Medical Centre, Rotterdam, Netherlands

**Keywords:** DNA repair, reporter, chromatin, protocol, non-homologous end-joining, microhomology-mediated end-joining, single-strand template repair, CRISPR

## Abstract

DNA double-strand breaks (DSBs) can be repaired through various pathways. Understanding how these pathways are regulated is of great interest for cancer research and optimization of gene editing. The local chromatin environment can affect the balance between repair pathways, but this is still poorly understood. Here we provide a detailed protocol for DSB-TRIP, a technique that utilizes the specific DNA scars left by DSB repair pathways to study pathway usage throughout the genome. DSB-TRIP randomly integrates a repair reporter into many genomic locations, followed by the induction of DSBs in the reporter. Multiplexed sequencing of the resulting scars at all integration sites then reveals the balance between several repair pathways, which can be linked to the local chromatin state of the integration sites. Here we present a step-by-step protocol to perform DSB-TRIP in K562 cells and to analyse the data by a dedicated computational pipeline. We discuss strengths and limitations of the technique, as well as potential additional applications to study DNA repair.

## 1 Introduction

The double-strand break (DSB) repair machinery consists of multiple pathways, including non-homologous end-joining (NHEJ), homologous recombination (HR) and microhomology-mediated end-joining (MMEJ) (McVey and Lee, 2008; Iliakis et al., 2015; Chang et al., 2017; Scully et al., 2019). Furthermore, a pathway named single-strand templated repair (SSTR) has been identified that can be utilized for templated CRISPR editing (Lin et al., 2014; Richardson et al., 2016). Many factors can influence which pathway is used to repair a specific lesion (reviewed in Brandsma and Gent, 2012; Ceccaldi et al., 2016; Scully et al., 2019). Several studies have demonstrated that the local chromatin state is one of the factors that influences which pathway is preferentially used to repair a DSB. These studies used methods ranging from using single imprinted endogenous loci (Kallimasioti-Pazi et al., 2018), single transgenic loci (Lemaitre et al., 2014), hundreds of endogenous loci (Iacovoni et al., 2010; Massip et al., 2010; Aymard et al., 2014) to thousands of integrated reporters as presented here (Gisler et al., 2019; Pokusaeva et al., 2021; Schep et al., 2021). All these studies rely on endonucleases creating a DSB at a defined locus in the genome, targeting either a definite sequence (e.g., restriction enzymes) (Iacovoni et al., 2010) or a user-defined one (Kallimasioti-Pazi et al., 2018; van Overbeek et al., 2016; Chakrabarti et al., 2019) [e.g., with CRISPR/Cas9 (Jinek et al., 2012; Cong et al., 2013; Jinek et al., 2013; Mali et al., 2013)]. While there are other methods to generate DSBs across the genome, such as ionizing radiation, non-ionizing radiation or chemically induced DSBs (reviewed in Vitor et al., 2020), they are less suitable to link chromatin state to DNA repair pathway usage.

The advantages of using a single endogenous or transgenic site are that these loci can be easily imaged and perturbed in a controlled manner [e.g., nuclear lamina targeting (Lemaitre et al., 2014)], which allows for precise dissection of their repair kinetics [e.g., imprinted loci (Kallimasioti-Pazi et al., 2018)]. However, this approach does not provide the diversity of sites that multiplexed assays offer. Working with single loci can also be much more labour intensive to collect sufficient data to dissect the effects of the large variety of chromatin states on DNA repair.

Other techniques use restriction enzymes cutting multiple endogenous loci. The I-PpoI endonuclease targets the ∼300 copies of the 28S ribosomal RNA gene plus 15 other unique sites. It was mainly used to understand the interplay between DNA repair and transcription, as well as histone distribution (Berkovich et al., 2007; Goldstein et al., 2013; Kim et al., 2016). The Legube lab developed the DSB inducible *via* AsiSI (DIvA) cell line expressing the AsiSI restriction enzyme (Iacovoni et al., 2010; Massip et al., 2010; Aymard et al., 2014). The enzyme is fused to a ligand-inducible domain for controlled nuclear localization and can reliably create ∼150 endogenous breaks in U2OS cells in an inducible manner. This method can accurately measure differences in repair pathway choice between transcribed and non-transcribed regions. Unfortunately, in this system, the cutting efficiency is in general very low in heterochromatin (Aymard et al., 2014). DNA repair can therefore not be accurately measured throughout this major chromatin type. Other limitations with these restriction enzymes are that the pool of target sites is fixed and that the varying sequence surrounding the target site might still affect the repair pathway balance.

Here we provide a detailed protocol of DSB-TRIP, a technology to measure the relative activity of multiple DSB repair pathways in many genomic locations with different chromatin states (Schep et al., 2021). DSB-TRIP is an adaptation of TRIP (Thousands of Reporters Integrated in Parallel), which was initially designed to measure the impact of chromatin context on gene regulation (Akhtar et al., 2013; Akhtar et al., 2014). The multiplexed nature of DSB-TRIP enables the probing of DSB repair hundreds or thousands of genomic locations, providing the statistical power needed to link differences in DSB repair pathway usage to a variety of chromatin features. We found that DSB-TRIP can detect DSB repair events across all chromatin states, including all known types of heterochromatin. Moreover, the design of DSB-TRIP effectively rules out confounding effects of surrounding DNA sequences.

### 1.1 Concept

**FIGURE 1:**
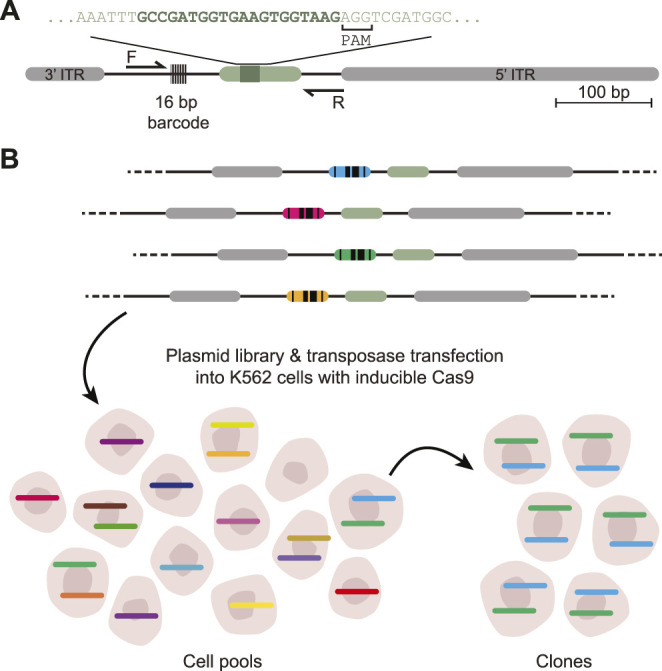
Scheme of DSB-TRIP. **(A)** Scheme of the barcoded DSB reporter. In grey the PiggyBac inverted terminal repeats, in light green the LBR endogenous sequence with the 20 bp gRNA sequence in dark green. Primers F and R are used to sequence the indels and the barcode. **(B)** Representation of the barcoded library and an illustration of the TRIP cell pools and clones with the different barcodes represented by different colours. Adapted from (Schep et al., 2021).

DSB-TRIP works by random integration of a specially designed DSB repair pathway reporter into hundreds or thousands of genomic locations in a pool of cells, by means of a transposon vector. The reporter is short [∼650 base pairs (bp)] and devoid of transcriptionally active sequences that could change the local chromatin environment (Figure 1A). Each copy of the reporter is marked by a random barcode, which allows for decoding of individual reporters and linking them to their genomic location. First, the genomic locations of the reporter integrations in the pool of cells are mapped. It is also possible to generate clonal cell lines that carry up to dozens of reporters. Next, a DSB is introduced inside each reporter by means of Cas9. Repair of the resulting DSBs results in specific “scars” [insertions and deletions (indels)] that can be used to identify the repair pathways that were active at a DSB (Allen et al., 2018; Chakrabarti et al., 2019; Chen et al., 2019; Shen et al., 2018; van Overbeek et al., 2016; Brinkman et al., 2018). Our current reporter can detect NHEJ, MMEJ and SSTR. After DSB induction and DNA repair, the genomic DNA is extracted and the reporter “scar” and the flanking barcode are jointly amplified by PCR and subjected to high throughput sequencing. A computational pipeline counts the scars for each individual barcoded reporter, infers from these counts the relative activity of each pathway, and links the results to the genomic location. Overlay with epigenomic mapping data then uncovers any correlations between pathway usage and local chromatin features.

A key feature of DSB-TRIP is that an identical reporter sequence (except for the short barcodes) is integrated into many different chromatin environments. Hence, differences in pathway usage between integration sites can be attributed to differences in the chromatin context. Thus, insights are obtained in the impact of the chromatin environment on DSB repair.

### 1.2 The Protocol in Brief

The protocol consists of two main components: the wet-lab experiments, and the computational analysis of the resulting sequencing data.

The wet-lab part starts with the design of the DSB repair reporter, which is a DNA sequence that, when repaired after a DSB, produces a specific indel pattern that can be associated with either NHEJ or MMEJ. We then describe how to make reporter plasmid libraries, how to transfect them into K562 cells and how to generate cell pools and clones carrying multiple integrations (Figure 1B). Next, two sets of data are generated: mapped reporter integrations and indel scores per reporter. First, the genomic location of the integrated pathway reporters (IPRs) in these clones are mapped by inverse PCR (iPCR), as explained in detail in (Akhtar et al., 2014). Second, the DSBs are induced by Cas9 induction, cells are cultured up to 72 h to allow the DSBs to be repaired, after which genomic DNA is collected to create sequencing libraries.

The sequencing data of the mapping and the indels are then processed with a computational pipeline that produces tables of the mapping per barcode and indels per barcode for further analysis.

The most basic implementation of the data analysis pipeline uses two types of sequencing data: iPCR reads used to link barcodes to the integration locations in the genome (mapping) and indel PCR sequences to assess repair outcomes. The standard output for the iPCR is a table containing the barcode, its genomic location (chromosome, start, end, and orientation), reads mapped, mapping quality and mapped sequence. The output for the indel PCR is a table containing four columns: the barcode sequence, the mutation class (e.g., wild-type, insertion, deletion or “unclear”), the size of the detected indel in bp and the number of occurrences. Two main extensions can be implemented for more detailed output. First, the pipeline can be run to count specific repair scars. This can be used to differentiate MMEJ from non-MMEJ scars with the same indel size. This functionality is available in the pipeline but requires a significantly longer runtime. The second option available, is the ability to add a recognition sequence to detect homology directed repair.

### 1.3 Choice of Cell Line

A prerequisite for DSB-TRIP is a cell line in which a DSB can be induced in a specific and reproducible way. We recommend establishing a clonal founder cell line with a stably integrated, inducible Cas9. We use K562 cells expressing Cas9 from the human PGK promoter and fused to a destabilization domain (DD) at its N terminus. This DD causes active degradation of the protein unless it is stabilized by addition of the small molecule Shield-I (Banaszynski et al., 2006; Brinkman et al., 2018). This allows for tight control of Cas9 activity. We note that in our cell line (clone K562#17) full induction of DD-Cas9 takes ∼16 h, which should be taken into account in the experimental design. Possibly, Cas9 can be introduced into cells by lentiviral transduction, or by transient transfection with an expression vector or Cas9 ribonucleoprotein. However, this may not yield homogeneous expression in 100% of the cells, which may compromise the data quality.

Instead of K562 cells, other cells may be used. However, there are some practical restrictions. First, the cell line must tolerate DNA transfections and transposon integrations; this is a requirement for the reporter insertions as well as for sgRNA transfections. Second, the cells must tolerate some levels of DNA damage, as some individual cells in the pool might carry more than 20 IPRs in their genome, and thus may potentially need to cope with as many simultaneous DSBs. Third, epigenome mapping data must be available for the cell line. This is essential for linking of the DSB-TRIP results to the chromatin state of the IPRs.

### 1.4 Designing the Reporter

One should consider the following elements when designing the reporter. First, a reporter of small size (≤1 kb) and devoid of any active transcription will have less impact on the chromatin state at the integration site. Second, the reporter sequence should produce signature “scars” after repair of the DSB that are characteristic of the repair pathway(s) of interest. We used a short sequence derived from the *LBR* gene (Schep et al., 2021), but it may be replaced by other sequences depending on the pathway of interest. Third, when designing the reporter sequence it is important to have the correct restriction sites at each end of the sequence (Section 1) as well as the *IndelPCR1_rv* primer binding site at the NheI end to be able to follow the library preparations steps (Section 13).

### 1.5 Limitations of DSB-TRIP

Despite the power of DSB-TRIP, it has some limitations. First, DSB-TRIP is based on the detection of signature indels that are produced by the respective pathways. However, HR does not usually generate indels, and hence this pathway cannot be measured with DSB-TRIP. The reporter that we present here is also sensitive to large deletions or extensive resection that might remove primer binding sites or the reporter barcode. In part this can be overcome by using Tn5 transposon-based library preparation as we presented in (Schep et al., 2021) or with UdiTaS (Giannoukos et al., 2018) which requires only one specific primer site. Second, while Cas9 has been the most prominent tool for gene editing, the DSBs that it creates may not be representative for DSBs that occur naturally. For example, the cutting and repair rates that have been observed with Cas9 appear to be extremely slow (Brinkman et al., 2018). This should be kept in mind while interpreting the data. Due to the slow rates of cutting and repair (a typical DSB-TRIP experiment takes 3 days), it is difficult to link observed pathway activities to cell cycle stages. Finally, in the cell pools the number of integrations per cell can vary substantially, which may lead to different DNA damage loads that in turn may affect the DNA repair kinetics or pathway balances.

### 1.6 Additional Applications of DSB-TRIP

While the generation of TRIP cell pools and clones can be time consuming there are many ways to use these TRIP cell pools and clones. The cell pools offer many different genomic loci, but note these experiments also require many cells to have sufficient coverage per IPR. For more multiplexed applications, such as small molecule or CRISPR screens, we recommend the use of clones carrying multiple IPRs. DBS-TRIP is compatible with experiments in 96- or even 384-well plates. This format is perfect for screens and other automated applications. For instance, a multiplexed, automated time series analysis in 96-well format was presented in Schep et al. (2021).

**FIGURE 2:**
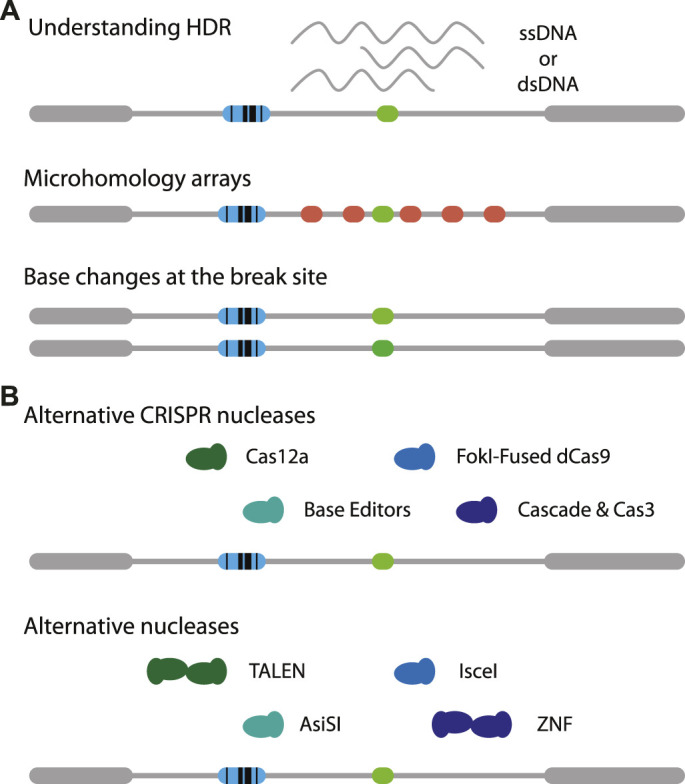
Adaptations of DSB-TRIP. **(A)** Potential variations of the reporter itself. One can study the effects of chromatin on (1, top) MMEJ by creating tiled microhomologies (red), (2, middle) on Cas9 mutagenesis with small mismatches (middle) or (3, bottom) on HDR based transgenesis. **(B)** Illustration on variations on CRISPR nucleases (top) and other nucleases (bottom). Target cut site in light green, IPR barcode in blue.

The reporter assay can be modified in many ways. For example, the DSB target site may modified with different microhomologies to study MMEJ in more detail. It is also possible to add variations in the target sequence to study how chromatin might affect slight changes in affinity of Cas9. This may provide a better understanding of off-target cutting by Cas9 and the ensuing repair (Figure 2A, middle). Using the exact same reporter as presented here it is feasible to study SSTR in the context of chromatin, by co-transfecting a single-stranded oligonucleotide that carries a small insertion (Schep et al., 2021). It may also be possible to provide different types of templates, for instance a double-stranded DNA template (Wienert et al., 2020). Furthermore, the effects of adding mismatches and varying the homology arm lengths may be investigated (Figure 2A, bottom) (Richardson et al., 2018; Hussmann et al., 2021). Potentially, time-series measurements on DSB-TRIP cell pools or clones combined with mathematical modelling (Brinkman et al., 2018) may reveal how rate constants of individual steps of the cut-and-repair process are affected by the local chromatin state.

It will be interesting to apply DSB-TRIP with other CRISPR endonucleases such as Cas12a (Zetsche et al., 2015) or rare-cutter restriction enzymes such as I-SceI (Niu et al., 2008) or AsiSI (Iacovoni et al., 2010) (Figure 2A, bottom). I-SceI was successfully applied to study Single Strand Annealing in a TRIP assay (Pokusaeva et al., 2021). Another option are transcription activator-like effector nucleases (TALENs) (Boch et al., 2009; Gaj et al., 2013; Moscou and Bogdanove, 2009), especially since it was found that these nucleases are more efficient in heterochromatin, compared to Cas9 (Jain et al., 2021). Furthermore, mismatch repair may be studied using base editors (Figure 2A, top) [reviewed in (Anzalone et al., 2020)]. For each of these endonucleases it is essential that they are expressed in a tightly controlled inducible manner, otherwise the DSBs are already generated and repaired while the cell pools with IPRs are being established.

Finally, it will be interesting to study chromatin context effects on DNA repair in cells with particular genetic alterations, such as mutations in specific repair proteins or defects in chromatin organisation, such as Hutchinson-Gilford Progeria that is the result of specific mutations in the Lamin A gene (De Sandre-Giovannoli et al., 2002; Eriksson et al., 2003). With its flexibility, multiplexing ability and detailed readout, DSB adds a versatile tool to study DNA repair, gene editing and the interplay between repair pathways and chromatin.

## 2 Step by Step Methods

### 2.1 Experimental Procedure

#### 2.1.1 DSB-TRIP Library Cloning

**FIGURE 3:**
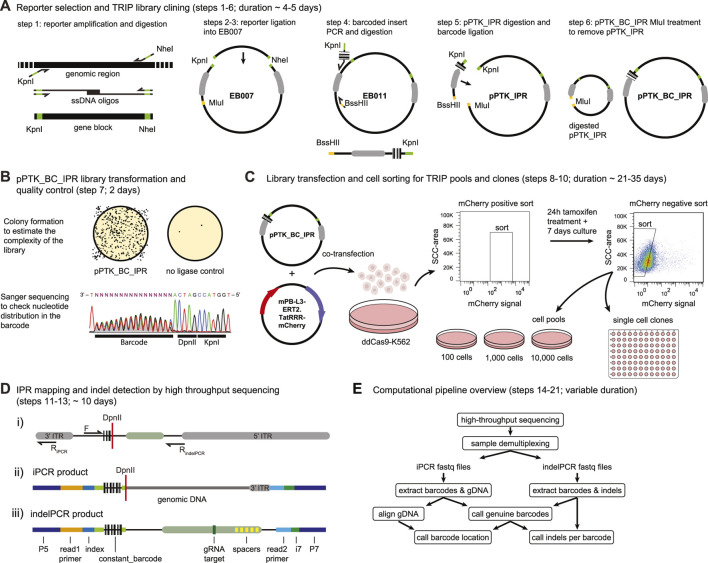
Step by step DSB-TRIP. **(A)** DSB-TRIP cloning steps. DNA sequences are in black, important restriction enzyme sites in green and orange and the PiggyBac ITRs are in grey. **(B)** Library quality control. Top: assessing the complexity of the library by counting the colonies. Bottom: A Sanger sequence track from step 7, spanning the barcode and the DpnII and KpnI restriction sites in the plasmid library, The sequence is flipped to match figure orientation. All the bases are equally present in the 16 bp barcode. **(C)** Library transfection and FACS sorting strategy to generate pools and clones. **(D)** i) Scheme of the IPR with the main primers used for generating the mapping (F & R_iPCR_) and the indel (F & R_indelPCR_) sequencing libraries. DpnII restriction site is indicated with a red line. ii) Scheme of a raw iPCR library PCR product, created from the circularisation of the fragment by ligation of a genomic DpnII site with the DpnII site from above. From left to right: P5 Illumina adapter, read1 primer site, 16 bp IPR barcode, DpnII restriction site in red, genomic DNA dark gray, 3′ITR in light gray, i7 Illumina index, P7 Illumina adapter. iii) Scheme of a raw indelPCR library PCR product, indicating the Illumina adapter and primer locations (P5, read1 primer, read2 primer, i7 and P7) as well as constant sequences related to the config file. From left to right: P5 Illumina adapter, read1 Illumina primer, index, constant_barcode (light green) with 16 bp IPR barcode embedded in between, LBR sequence containing the Cas9 target site (dark green) and multiple spacers in yellow (spacer_list), read2 Illumina primer, i7 Illumina index, P7 Illumina adapter. **(E)** Diagram of the analysis pipeline.

NOTE: Can be done in parallel with Section 2.

1. Preparation of the reporter insert (Figure 3A)—30 min

**Table:** 

Component	Amount (µl)	Final concentration
10× CutSmart buffer	5	1×
0.1–1 µg insert	a	
NheI-HF (20 U/µl)	1	0.4 U/µl
KpnI-HF (20 U/µl)	1	0.4 U/µl
Nuclease-free water	43—a	
Total	50

1.1. Digest 100 ng—1 µg of the insert (amplified by PCR, from oligos, or as a geneblock—i.e., gBlocks™ from IDT) with the following mix:
1.2. Incubate 10 min at 37°C
1.3. Purify the digested product with a PCR purification kit, following the manufacturer’s instructions. Kits such as PCR Isolate II PCR and Gel Kit (Bioline) or CleanPCR beads (cleanNA) can be used for any PCR purification steps in this protocol.
1.4. Measure the insert concentration by nanodrop.
 2. Preparation of the TRIP vector (Figure 3A)—30 min
2.1. Digest 100 ng of the plasmid EB007 as described above.
2.2. Dephosphorylate the ends using either Quick CIP (NEB #M0525), Shrimp Alkaline Phosphatase (rSAP) (NEB #M0371) or Antarctic Phosphatase (AP) (NEB #M0289) and heat inactivate the enzymes, using the supplied protocol.
 3. Ligation and Transformation of the reporter into the TRIP vector (Figure 3A)—1.5 days

NOTE: This can be done in parallel with the barcoded insert preparation—Section 4).

**Table:** 

Component	Amount (µl)	Final concentration
10× T4 DNA ligase buffer	2	1×
50 ng reporter insert from step 1.4	a	
Digested EB007 from step 2.2	1	0.3 U/µl
T4 DNA Ligase (5 U/µl)	1	0.3 U/µl
Nuclease-free water	16—a	
Total	20	

**Table:** 

Component	Amount (µl)	Final concentration
5× Phusion high-fidelity buffer	100	1×
5 ng EB011	a	10 pg/μl
barcoding-primer-fw (100 µM)	2	0.4 µM
barcoding-primer-rv (100 µM)	2	0.4 µM
dNTP mix (10 mM)	10	0.2 mM
Nuclease-free water	376—a	
Phusion DNA polymerase (2 U/µl)	10	0.04 U/µl
Total	500

3.1. Mix 50 ng of the insert with 1 μl of digested EB007, 1 μl of T4 DNA ligase (Roche Cat#: 10799009001) and 2 μl T4 DNA Ligase Buffer in 20 μl final volume.
3.2. Incubate 10 min at RT (room temperature).
3.3. Transform 2 μl of the ligation reaction into JM109 competent cells together with a no-ligation control.
3.4. Pick 10 colonies and grow them in 2 ml LB with 100 μg/ml ampicillin for 8 h, purify the plasmids using PureLink™ HQ Mini Plasmid DNA Purification Kit (Thermo—or similar) and quantify using a nanodrop.
3.5. Verify the correct plasmid sequence with Sanger sequencing using primer *barcode-sanger-rv*.
*barcode-sanger-rv* | TAC0005 | CGCCAGGGTTTTCCCAGTCACAAG.
3.6. Expand the selected mini culture in 100 ml LB with 100 μg/ml ampicillin and purify pPTK_IPR with PureLink™ HiPure Plasmid Midiprep Kit (or similar).
3.7. Measure pPTK_IPR concentration by nanodrop.
 4. Preparing the barcoded insert (Figure 3A)—3 h
4.1. Prepare the following PCR mix to make the barcoded insert. Split the total volume in five 100 µl reactions.

NOTE: Do not use EB007 here as it has a point mutation in the 3′ITR of the PiggyBac transposon.

**Table:** 

Cycle number	Denature	Anneal	Extend
1	95°C 1 min		
2–26 (25 cycles)	95°C 30 s	58°C 30 s	72°C 30 s
27			72°C 1 min

**Table:** 

Component	Amount (µl)	Final concentration
10x CutSmart buffer	20	1x
pPTK_IPR from step 3.7	A	
BssHII (5 U/µl)	12	0.3 U/µl
KpnI-HF (20 U/µl)	3	0.3 U/µl
Nuclease-free water	165—a	
Total	200	

**Table:** 

Component	Amount (µl)	Final concentration
10× CutSmart buffer	20	1×
pPTK_IPR from step 3.7	a	
MluI-HF (20 U/µl)	3	0.3 U/µl
KpnI-HF (20 U/µl)	3	0.3 U/µl
Nuclease-free water	174—a	
Total	200	

**Table:** 

Component	Amount (µl)	Final concentration
Nuclease-free water	16—a—b	
10× T4 Ligase buffer	2	1×
Purified KpnI-MluI-digested pPTK_IPR vector	A	∼15–25 ng/μl
Purified KpnI-BSSHII digested barcoded insert	B	molar ratio of 1:5 vector:insert
T4 DNA ligase (5 U/µl)	2	0.5 U/µl
Total	20	

**Table:** 

Component	Amount (µl)	Final concentration
10× CutSmart buffer	5	1×
Purified pPTK_BC_IPR from step 6.4	43	
MluI (10 U/µl)	2	0.4 U/µl
Total	50

**FIGURE 4:**
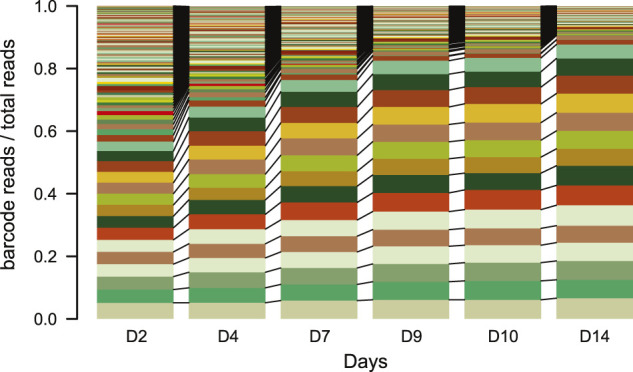
IPR expansion and drift over time. Stacked barplot of the percentage of reads each barcode represents over time (days). Each color represents a different barcode (IPR) that are linked between the plots *N* = 1. Over the time course of the experiment samples were taken from a TRIP cell pool and processed as for scoring indels. The barcodes were retrieved from the indelPCR data, counted and their proportion was plotted per day.

NOTE: Sequencing the whole pool without damage induction might help understanding the total complexity (number of different barcodes) and the barcode distribution. If the barcode distribution is strongly skewed toward more abundant ones, we recommend increasing the starting material during the library prep as well as increasing the sequencing depth. This will have to be optimized for every specific library and experimental setup.

4.2. Amplify using the following PCR program:
4.3. Pool the PCR tubes and run 5 µl on gel, a single band of 200 bp should appear.
4.4. Purify the PCR product with a PCR purification kit, elute in 100 µl nuclease-free water and quantify by nanodrop. Aim for a yield of ∼ 6–7 µg.
4.5. Digest the barcoded PCR product with KpnI and BssHII.
4.6. Incubate at 37°C for 2 h.
4.7. Purify with a PCR purification kit and elute in 30 µl nuclease-free water and quantify by nanodrop. Aim for a yield of ∼4–5 µg.
 5. Digestion of the pPTK_IPR for the barcode ligation (Figure 3A)—4 h
5.1. Digest the pPTK_IPR (step 3) with KpnI-HF and MluI-HF in the following reaction:
5.2. Incubate at 37°C for 2 h.
5.3. Purify with a PCR purification kit and elute in 88 µl nuclease-free water.
5.4. Dephosporylate and heat inactivate all of the digested pPTK_IPR using either Quick CIP (NEB #M0525), Shrimp Alkaline Phosphatase (rSAP) (NEB #M0371) or Antarctic Phosphatase (AP) (NEB #M0289) using the supplied protocol.
5.5. Purify the vector with a PCR purification kit, elute in 30 µl nuclease-free water and quantify by nanodrop. Aim for yield of ∼1.5–2 µg.
 6. Ligation of the pPTK_IPR with the barcoded insert (pPTK_BC_IPR) (Figure 3A)—1.5 days
6.1. Prepare the ligation mix on ice as well as a control reaction without insert. The control should be processed in parallel with the real TRIP library until step 7.8.
6.2. Incubate at 16°C overnight.
6.3. Add 80 µl nuclease-free water and heat-inactivate T4 DNA ligase for 10 min at 65°C.
6.4. Purify with a PCR purification kit and elute in 43 µl.
6.5. Prepare a digestion mix to digest any remaining original non barcoded vector:
6.6. Incubate for 1 h at 37°C
6.7. Purify the digestion with a PCR purification kit and elute in 50 µl nuclease-free water. NOTE: Include one extra wash step to make sure all remaining salt is removed. Small traces of salt can hinder the subsequent electroporation into bacteria.

NOTE: In case of issues with electroporation, extra bead purification using magnetic beads such as CleanPCR beads eluted in 10 µl can be used.

NOTE: It is important to elute in nuclease-free water, and not TE or EB as the sample might need to concentrated by SpeedVac in step 6.9 and high salt concentrations can affect the electroporation in step 7.1.

6.8. Measure the concentration of DNA by Qubit or Nanodrop spectrophotometer.

NOTE: The total yield should be about ∼250–400 ng of DNA.

6.9. Concentrate the DNA in a SpeedVac to reach a concentration of ∼50–200 ng/μl in a minimum of 5 µl.

NOTE: the pPTK_BC_IPR DNA can be stored at −20°C indefinitely.

7. Transformation of bacterial cells and preparation of the DSB-TRIP library (Figure 3B)—1.5 days
  7.1. Electroporate the pPTK_BC_IPR into CloneCatcher DH5α electrocompetent *E. coli* (Genlantis) using an Electroporation machine (Gene Pulser—Bio-Rad or similar) setup: Capacitance = 25 -Set volts to 2.00 (kV)–400 Ω resistance.

NOTE: It is essential that the *E. coli* are highly electrocompetent to generate a high complexity TRIP library. This would be a good starting point for trouble shooting any issues with getting high enough complexity in the libraries.

NOTE: The time constant should be >4 ms. A lower time constant is most likely the result of an arch discharge (spark) and might result in a lower TRIP complexity.

7.2. Immediately add 2 ml recovery medium (that comes with the electrocompetent cells) to the cells and let the cells recover for 30 min at 37°C on a shaker at 400 rpm.
7.3. To estimate the complexity of the pool, make three dilutions the transfected cells using a small fraction of the cells (20 µl) and plate these in three different LB agar plates containing 100 μg/ml ampicillin. We recommend final dilutions of 1:1,000, 1:10,000 and 1:100,000. Incubate these plates at 37°C O.N. Repeat this with the no insert negative control.

NOTE: This will be used to estimate the complexity of the library, count the number of colonies per plate and multiply that by each dilution factor. This will typically range between 1 and 10 × 10^6^, with at least 2–3 × 10^5^. The control plate should have 50–100 times less cells, indicating minimal contamination (1–2%) of the initial vector.

7.4. Transfer the remaining cells into a sterile flask with 200 ml of LB medium containing 100 μg/ml of ampicillin, incubate at 37°C O.N. with vigorous shaking.
7.5. Collect the cells from the previous step and pellet them by centrifugation at 5,000 *g* for 15 min at 4°C and keep them on ice.
7.6. Purify the plasmid DNA using a plasmid maxi kit following the manufacturer’s instructions. Elute the pPTK_BC_IPR library in 600 µl of nuclease-free water.
7.7. Measure the concentration with a Nanodrop spectrophotometer. Standard yield is about 1 μg/μl. NOTE: This plasmid library can be stored at −20°C indefinitely.
7.8. Verify the correct distribution of nucleotides by Sanger sequencing the plasmid library using primer *indelPCR1-rv*, following the recommendations of the Sanger sequencing service used.

NOTE: It is important to see an equal distribution of the nucleotides at the position of the barcode. If the distribution of the four nucleotides is not equal this might mean that the barcode library is biased and might not be as complex as expected (Figure 3B).

#### 2.1.2 Creating DSB-TRIP Cell Pools and Clones

8. Transfection of K562-DD-Cas9 cells (Figure 3C)—2 h
  8.1. Culture a fresh batch of K562-DD-Cas9 in complete RPMI, expand the cells to reach at least 20 million cells for the transfection.
  8.2. Plate 9 million cells per plate in 2 10-cm dishes. One plate will be used for the actual library, the second will serve as a mock transfection.
    NOTE: For the mock transfection replace the mPB-L3-ERT2. TatRRR-mCherry plasmid with nuclease-free water.
  8.3. Prepare the lipofection DNA dilution in OptiMEM as follows:

**Table:** 

Component	Amount (µl)	Final concentration (ng/µl)
TRIP library (30 μg; from step 7.7)	*X*	20
mPB-L3-ERT2.TatRRR-mCherry plasmid (5 μg)	*Y*	4
Opti-MEM medium	1,500	

**Table:** 

Component	Amount (µl)	Final concentration
Lipofectamine 2000	*60*	4%
Opti-MEM medium	1,500

8.4. Prepare the lipofectamine dilution in OptiMEM as follows:
8.5. Incubate both dilutions for 10 min at RT.
8.6. Mix the solutions together and flick the tube to mix the lipofection solution. Incubate for 20 min at RT.
8.7. Add the lipofection solution dropwise to the plates. Incubate the cells at 37°C overnight. Refresh the medium the next morning by centrifuging the cells at 300 g for 5 min at RT and resuspending them in fresh complete RPMI medium.
8.8. Asses the transfection efficiency (mCherry signal) after ∼18 h under a fluorescence microscope. The proportion of mCherry-positive cells should be at least 10%.
 9. Sorting of the transfected cells (Figure 3C)—7 days
9.1. After 18–30 h of transfection, prepare the cells for FACS sorting. Transfer the cells into a 15-ml conical tube and centrifuge for 4 min at 300 g at RT.
9.2. For the transfected cells, aspirate the medium and resuspend the cells in 1.5 ml of PBS supplemented with 1% FBS. Place the cells in a FACS tube on ice and head to the sorting machine.
9.3. Right before the sort, pass the cells through a cell-strainer cap on a FACS tube and install the tube in the machine.
9.4. For the sorting strategy follow step 52 [Nature Protocols (NP):52] in (Akhtar et al., 2014).
9.5. Centrifuge the sorted cells for 4 min at 300 g at RT. And resuspend the cells in warm fresh RPMI medium at approximately 10^5^ cells per ml containing 0.5 µM 4-hydroxytamoxifen (4-OHT) to activate the transposase.
9.6. Culture the cells for 24 h and then wash the cells by centrifugation at 300 *g* for 4 min at RT and resuspend them in fresh complete RMPI medium to avoid rehopping of the transposon.
9.7. Continue culturing the cells for ∼5–7 days and expand the culture conditions if they become too confluent. Aim for at least 5× the plasmid library complexity (step 7.3) by the end of this period for generating the TRIP pools and clones.

NOTE: This period is required do get rid of the free-floating plasmids in the cells that might cause background rehopping of the transposon.

10. Generate TRIP cell pool and clones. (Figure 3C) >7 days
  10.1. Passage the cells one final time 24 h at a density of 2–5 × 10^5^ cells per ml before the second sort to make TRIP cell pools. This is needed to make conditioned medium for the TRIP cell pools and clones for a higher chance of recovery.

NOTE: We recommend collecting 12 ml conditioned medium per 96-well plate and 25 ml for a full 24 well plate for the cell pools.

10.2. To prepare the cells for the FACS sort to make TRIP cell pool, collect the cells and pellet them by centrifugation at 300 *g* for 4 min at RT. Collect the medium from the cells and filter it through a 20 µm sterile filter. Prepare the collection plates. Distribute 1 ml per well in a 24-well plate for the pools and 100 µl per well in two 96-well plates for the clones. Keep the plates at 37°C until the sort.
10.3. Resuspend the cells in PBS supplemented with 1% FBS and place them on ice until the sort. Right before the sort, pass the cells through a cell-strainer cap on a FACS tube and install the tube in the machine.
10.4. Sort live cells to generate pools with different starting number of cells, this will determine the complexity of the pool. The type of following experiment will determine if higher or lower complexities are required. Therefore, we recommend sorting cells in pairs with different starting number (500–10,000).
10.5. Sort single live cells into the 96-well plates to directly generate clones.

NOTE: in our experience, we obtain ∼10–20 fit and healthy clones from one 96-well plate.

10.6. Collect the left-over cells as a backup, culture them for one more day to recover and cryo-store them in complete RPMI medium +10% FBS.
10.7. Expand the TRIP cell pools and clones and prior to cryo-storage collect ∼10^6^ cells for integration counting and mapping.

NOTE: It can take more than 3 weeks before the clones reach at least 10^6^ cells and they might grow at different rates.

10.8. To measure the average number of integrations per cell, we suggest two options:
 a) By qPCR: follow the Box 2 in (Akhtar et al., 2014).
 b) If clonal lines were generated from the TRIP cell pool, the number of integrations can be estimated by high throughput sequencing of barcodes in each clone. When clones are randomly selected, these clones should give a fair representation of the number of integrations per cell.
 11. Mapping the IPRs (Figure 3D, i,ii)—5 days
11.1. Collect genomic DNA (gDNA), by using a standard genomic DNA extraction kit, from TRIP cell pools and/or clones to obtain about 1 µg of gDNA for the clones and 6 µg of gDNA for the pools.
11.2. For the mapping of the pools and the clones by inverse PCR, follow steps NP:83–105 from (Akhtar et al., 2014) with the following modifications:
  a) We recommend setting up the DpnII digestion twice (step NP:87) in case of low yield after purification or a failed PCR step.
  b) The ligation purification by precipitation at step NP:89–91 can also be done by bead purification at a beads:sample ratio of 1.5:1 following the manufacturer’s instructions.

#### 2.1.3 DSB Induction and Repair

NOTE: The required number of cells will differ depending on the complexity of TRIP integrations in the cell pools. Aim for at least 1,000× of the pool’s starting number of cells prior to nucleofection. This aims to correct for cell death after nucleofection and to have sufficient coverage per IPR for the library preparation. E.g., 2 × 10^6^ cells for a cell pool of 2000 starting cells (TRIP-2000 pool).

NOTE: Do not culture the pools for too long. After a lot of culturing, the complexity of the TRIP cell pools can decrease as fast cycling cells will take over most of the reads in the pools within two to 3 weeks (Figure 4).

12. sgRNA plasmid transfection, Cas9 activation and cell collection—3 days

For the K562-DD-Cas9 TRIP-2000 pool:

12.1. Collect all the cells from a high density 10 cm dish in a 15 ml falcon and count the cells using a cell counter.
12.2. Transfer four million cells into a new 15 ml falcon and centrifuge at 300 g for 4 min at RT. Two million for the treated cells and 2 million for the no-guide transfection control.
12.3. During this time prepare the transfection buffer: add 8 µl of 100 mM ATP to 100 µl of homemade Amaxa buffer (reagent setup) per sample.
12.4. Prepare two Eppendorf tubes for the plasmids to transfect. Add 2 µg of sgRNA plasmid to one tube and 2 µg of GFP expressing plasmid to the other tube.

NOTE: The quantity of sgRNA plasmid can be optimized by transfecting different amounts of sgRNA plasmid and checking for indel frequencies by TIDE in the parental cell line or one of the TRIP cell pools while the other clones and pools are growing.

12.5. Remove the supernatant and resuspend the cells in 200 µl of complete transfection buffer.
12.6. For each transfection reaction, take 100 µl of cell suspension and mix it with the plasmid in the tube and directly transfer the whole volume into a nucleofection cuvette.
12.7. Nucleofect the cells with program T-016 on an Amaxa 2D Nucleofector.
12.8. Immediately add 900 µl of complete RPMI medium to each cuvette. And then, using a small Pasteur pipet, transfer the cells to a new 10 cm dish with fresh complete RPMI.
12.9. Let the cells recover from the transfection for ∼16 h.
12.10. Add 500 nM Shield-1 to the cells to activate Cas9.
12.11. Mix by swirling the plate and place the plate in the incubator.
12.12. Collect 2 × 10^6^ cells in a 15 ml falcon at the desired time points, typically 72 h for the endpoint of the cutting and repair reaction.
12.13. Centrifuge the cells at 300 g for 4 min at RT and remove the supernatant.
12.14. Extract gDNA by using a genomic DNA extraction kit following the manufacturer’s instructions.

#### 2.1.4 High Throughput Sequencing Libraries

13. Library preparation for the indel detection (Figure 3D, iii)—1 day

NOTE: It is advised to check for total indel frequency by *TIDE* prior to library preparation (Brinkman et al., 2014; Brinkman and van Steensel, 2019).

**Table:** 

Component	Amount (µl)	Final concentration
gDNA—200–500 ng	*x*	4–10 ng/μl
MyTaq™ Red mix 2×	25	1×
indelPCR1-fw-indexed 10 µM	0.5	100 nM
indelPCR1-rv 10 µM	0.5	100 nM
Nuclease-free water	24—*x*	
Total	50

13.1. Prepare the indelPCR1 mix as follows:

NOTE: A complex pool will require more starting material than clones or less complex pools. Considering a mainly triploid K562 genome (Zhou et al., 2019), 100 ng of gDNA represents about 10^4^ K562 cells. For complex pools aim for at least a 50× coverage of the starting number of cells (50 × 2,000 = 10^5^ cells; so ∼1 µg of gDNA). This should be sufficient to recover most of the IPRs in a reproducible manner as we account for some cell death during the pool expansion (not all 2,000 cells will survive) and during standard culturing. In our case we will run this in triplicates to increase the coverage of our TRIP-2000 pool.

NOTE: The indexed PCR primers allow sample multiplexing for next generation sequencing. The indices from the indelPCR1-fw-indexed primer count as internal barcodes and will not be automatically demultiplexed by the Illumina sequencer.

NOTE: Important considerations to take when mixing indices. Pick a varied set of indices, if possible, avoid mixing indices with only a couple of mismatches. Avoid indices starting with or containing two guanines (Gs) in a row, especially for NovaSeq and NextSeq machines from Illumina. The Gs are not illuminated in these machines and therefore are prone to mistakes if they are at the start of the read.

**Table:** 

Cycle number	Denature	Anneal	Extend
1	95°C 1 min		
2–4 (3 cycles)	95°C 15 s	58°C 15 s	72°C 10 s
4–13 (10 cycles)	95°C 15 s	68°C 15 s	72°C 10 s
14			72°C 1 min

**Table:** 

Component	Amount (µl)	Final concentration
indelPCR1 product	*5*	
MyTaq™ Red mix 2×	25	1×
indelPCR2-fw 10 µM	0.5	100 nM
indelPCR2-rv-indexed or indelPCR2-rv 10 µM	0.5	100 nM
Nuclease-free water	19	
Total	50

13.2. Run the indelPCR1 with the following conditions.
13.3. Prepare the indelPCR2 mix as follows:

NOTE: Depending on the complexity of the library, indelPCR2-rv can be used with or without an index (i7).

**Table:** 

Cycle number	Denature	Anneal	Extend
1	95°C 1 min		
2–4 (3 cycles)	95°C 15 s	58°C 15 s	72°C 10 s
4–13 (10 cycles)	95°C 15 s	70°C 15 s	72°C 10 s
14			72°C 1 min

13.4. Run the indelPCR2 with the following conditions
13.5. Run 10 µl of indelPCR2 on a 1% agarose gel, a band should be visible around 280 bp.
13.6. Pool ∼10 µl of the samples based on the band intensity to approximately match the concentrations.
13.7. Purify the library by bead purification following the manufacturer’s instructions at a bead:sample ratio of about 0.8:1, to keep only fragments >200 bp.
13.8. Measure the concentration using a Qubit dsDNA HS Assay Kit on a Qubit Fluorometer.
13.9. Sequence the library with the aim to obtain about four million reads per pool, single-end, 150 bp read length. NOTE: For a TRIP cell pool it is recommended to aim for at least a median 2000 reads per IPR per sample. For a trip clone, ∼500 reads per IPR per sample is sufficient.

### 2.2 Bioinformatics Pipeline

#### 2.2.1 Setting up the Environment for the Snakemake Pipeline (Figure 3E)

14. Downloading the scripts—10 min

NOTE: all code below is run in the *linux* shell from the terminal.

14.1. To successfully run the *Snakemake* pipeline we recommend to download the DSB-TRIP *github* repository (https://github.com/vansteensellab/DSB_TRIP_protocol):

15. Setting up the conda environment—variable
  15.1. Install the *conda* environment ‘DSB_TRIP_env.yml’ that is supplied with the repository

15.2. Activate the *conda* environment

16. Download extra dependencies to lib folder—10 min

17. Generating Bowtie index for reference the genome—2 h
  17.1. Download the reference genome of interest (e.g., hg38).
  17.2. Build a *Bowtie* index:

18. Configure the pipeline—10 min
  18.1. The example configuration file provided in the *GitHub* repository can be adapted to fit the users’ specific requirement. This could be a matter of changing some local paths (e.g., the path to the *Bowtie* index).

#### 2.2.2 Running the Pipeline

19. Running the Snakemake pipeline—1–4 h (depending on the amount of data and number of cores used)
  19.1. Run the DSB_TRIP *Snakemake* pipeline with 20 cores:

20. Calculate pathway balance and link to IPR location in genome (detailed below in the *Results* section)—1 h
  20.1. Select barcodes with unique location
  20.2. Filter out lowly abundant barcodes
21. Downstream analysis (detailed below in the *Results* section)—8 h
  21.1. Calculate pathway balance measures (e.g., ratio’s/proportions)
  21.2. Correlate the pathway balance measure of interest with chromatin information available (e.g., chromatin states/ChIP signal). This last step is outside the scope of this paper.

git clone https://github.com/vansteensellab/DSB_TRIP_protocol

conda env create -f config/DSB_TRIP_env.yml

source activate dsb_trip

./download_dependencies.sh

bowtie2-build </path/to/genome.fa.gz> </path/to/index>

snakemake –s src/dsb_trip.snake \--configfile <path/to/config.yaml> \--use-conda -j 20

## 3 DSB-TRIP Pipeline

The main functionality of the DSB-TRIP pipeline is to extract the integration site for every barcode from the iPCR data, and the indel data for every barcode from the indel PCR. With the correct configuration it is able to give easily interpretable results.

### 3.1 Pipeline Configuration

The DSB-TRIP pipeline is written as a *Snakemake* pipeline (Molder et al., 2021). Two files are required for successful execution: 1) a meta-data file with information on the samples, and 2) a pipeline configuration file with pipeline parameters.

1. Meta-data file

A tab-delimited file with information on the samples: sample name, file location, type of PCR (e.g., iPCR, indelPCR), and plasmid guide information.

2. Configuration file

The configuration file determines the DSB-TRIP pipeline execution. Parameters include but are not limited to: description of the read structure, thresholds used in the pipeline, information on the reporter for DNA scar analysis, and alignment parameters.

### 3.2. Pipeline Process

The DSB-TRIP pipeline consists of several modules which will perform the following four tasks: pre-processing of the sequencing reads, barcode clustering, genomic alignment of the iPCR reads and scar analysis of the indel PCR reads.

1. Barcode retrieval

First, the barcode is extracted from iPCR and the indel reads. This implementation is based on *Cutadapt* (version 1.11) (Martin, 2011) and custom scripts. For both read types, a 16 bps barcode is positioned within a constant 5′ adapter sequence: “GTCACAAGGGCCGGCCACAA{barcode}TGATC”. The barcode is extracted using a three-step strategy: 1) matching of the entire sequence, 2) matching of the 5′ sequence, 3) matching of the 3’ sequence. This approach ensures accurate retrieval of barcodes shorter or longer than 16 bps (15 or 17 bp barcodes can be present). Only reads with successful barcode retrieval are selected for further processing. This barcode retrieval is performed on read 1 of the iPCR paired-end sequencing reads. An additional constant sequence is required for read 2 of the pair. Finally, the reverse complement of these constant patterns are removed from the 3′ end of the reads if present due to read-through into the backbone.

2. Barcode clustering

The retrieved barcode sequences are classified as “genuine” or “mutated” using the *starcode* clustering algorithm (Zorita et al., 2015). Barcodes are clustered based on Levenshtein distance (<2) and the most abundant barcode in the cluster is considered the “genuine” barcode. Currently this step is used as a filtering step, but alternatively, barcodes can be rescued by assigning all information from the mutated barcodes to the genuine barcode.

3. Reporter alignment

Remaining iPCR sequences are aligned to the human genome with *Bowtie 2* (Langmead and Salzberg, 2012). This is carefully done to deal with potential chimeric reads created by the circular ligation [as shown in (Akhtar et al., 2014) Supplementary Figure 1]. For such reads, two pieces of genomic DNA are ligated at an unknown junction. Briefly, to remove these events, two sequential alignment steps are performed. First, the read pairs are mapped independently and the mates reading directly into the genome from the edge of the transposon are filtered based on potential soft-clip at the 5’ end. These reads are either discarded, or if >16 bps soft-clipping occurred, the clipped sequence is extracted and realigned to the genome in a second alignment step. This ensures that in case of chimeric reads, the genomic sequence found next to the integrated reporter is aligned instead of some distant genomic sequence that got inserted at the circular ligation step. Reads that pass the quality thresholds are aggregated based on their barcode sequence. For every barcode, read counts and mapping quality for the two locations with most supporting reads are returned. This allows custom filtering to select trustworthy integration sites.

4. DNA scar analysis

The DNA scar analysis tests for two points: the presence of the wild-type sequence and the size of a possible indel compared to the original sequence. First, reads are matched against known recognition sequence(s), made up of the wild-type sequence and optionally, any number of expected introduced mutations. Second, the location of specific downstream sequences is compared with their location in the wild-type fragment to calculate an indel size. In our case, these are seven 6 bps sequences that are originally are positioned between 83 and 124 bps from the start of the sequence (Figure 3D, iii). This approach allows us to pick up the complete range of deletion sizes that can be captured by the PCR performed and deal with potential read errors or mutations downstream of the break site.

When a recognition sequence is found, the call will be named after the recognition sequence (“wt” for wild-type sequence, or in case of a directed mutation, the name given in the *configuration file*). When no recognition sequence is found, the call depends on the indel size, this call will be “del”, “wt_point_mut” or “ins” for indel sizes *<0*, *0*, *>0*, respectively. When no recognition sequence or indel size can be calculated, the call will be “not_clear”. The indel size will be calculated and reported even if in the first step a recognition sequence was found. This way, some reads might be given a “wt” call, but an indel size other than 0 (e.g., −1). This can occur due to either a technical artefact or biological mutation outside of the Cas9 targeted mutagenic event of interest.

Two alternatives to this scar analysis are implemented: 1) pairwise alignments that capture the exact DNA scar, 2) direct comparisons of the DNA scars with expected scars as predicted by *inDelphi* or *FORECasT* (https://partslab.sanger.ac.uk/FORECasT) (Allen et al., 2018; Shen et al., 2018). Both of these options will allow more fine separation of repair scars at the cost of significantly longer processing time. This allows for more detailed analysis such as separating MMEJ scars from NHEJ scars that resulted in the same size deletion.

## 4 Results

### 4.1 Pipeline Output

The DSB-TRIP pipeline creates various output files and log files with details on pipeline execution. Output is organized following the four steps described previously.

1. Barcode retrieval

The statistics file for every sequencing sample is produced and includes for each read all the intermediate steps in the barcode retrieval analysis. This information is useful to identify and solve read parsing issues.

2. Barcode clustering

The barcode clustering returns a table with three columns containing barcodes: one column with clustered barcodes, the second with the most prominent within the cluster (we call this the “genuine” barcode), and one column with the total number of occurrences of barcodes within the cluster. The two columns containing barcodes are used to filter intermediary files for iPCR and indel reads that only the rows with “genuine” barcodes are left. The total cluster count is ignored by the pipeline, but can be used to assess how well this barcode clustering performed.

3. Reporter alignment

For each iPCR sample two tables are generated (we call these the “mapping tables”) containing barcode locations and quality measures, one for each mate of the paired end reads. The P7 read (read 2, file ending in “2.table”) is most important since that read contains the part of the sequence starting at the end of the transposon (Figure 3D, ii). The P5 read (read 1, file ending in “1.table”) is partly made up of the barcode and surrounding transposon sequence, there is still some genomic DNA left in the sequence which should be aligned at a nearby GATC side where DpnII cut the DNA before circularization. The table of this mate in the read pair can be used as quality control.

4. DNA scar analysis

The “.count” files generated by the scar analysis contain a four column table with the total number of reads per combination of barcode, mutation call and indel size produced by step 4 of the pipeline (see above).

### 4.2 Pipeline Evaluation

Finally, the various pipeline output files can be used to evaluate the DSB-TRIP experiment. We advise several quality controls and downstream filtering steps, as described below:

- The first thing to do is to select the barcodes which give an accurate and unique location. We advise to select all barcodes which are supported by at least 5 iPCR reads with an average mapping quality larger than 10 at the primary location, having at least 95% of the reads located at this locus, with not more than 2.5% of the reads at a secondary location. These ratio’s and averages can be calculated from the read counts and sum of mapping quality found in the “mapping table” (file ending in “.2.table”, see above). This will remove barcodes with multiple alignment locations either because of technical reasons such as repetitive sequences or because a single barcode is integrated in multiple locations.
- The second step to see if the pipeline was correctly configured and that the data is of good quality is to check whether repair scars were correctly identified. There are three sequence calls which can be used as indicators for bad quality/incorrect pipeline configuration: “not_clear” calls, “wt_point_mut” calls and wild-type (“wt”) calls with non-zero indel size. All these calls should be minor, since they indicate situations which are not expected to result from normal repair events. The “not_clear” calls include reads that failed both the recognition sequence search as well as the indel calculation. The “wt” calls are expected to be made for reads of indel size 0, therefore, “wt” calls with non-zero indel size and indel size of 0 without “wt” call (“wt_point_mut”) should be rare.
- We recommend to filter out the “not_clear” calls. In previous analysis we had a mean of 17.3% (95% CI: 16.7–17.8%) of “not_clear” calls that were filtered out this way. In this analysis we left in the “wt_point_mut¨ and wild-type calls that didn’t have an indel size of 0. A different option is to remove these from further analysis. However, the previous evaluation step should have indicated these events to be of minor impact on the resulting indel ratios.
- After initial filtering, when working with a TRIP cell pool, barcodes can be filtered on abundance. Since barcodes in a pool are found in a limited number of cells, it is important to assess whether a barcode is represented by enough cells to obtain an accurate measurement. One way to filter for abundance is to estimate, for each barcode, the number of cells in which this barcode can be found. In (Schep et al., 2021) this was done by assuming that, for each experiment, there are approximately 100,000 cells (based on the amount of DNA used) and every cell has on average 6 barcodes (based on the clonal selection). For each barcode an approximate cell number can be calculated summing up its count for all mutation calls, dividing over the total count of the experiment and multiplying this by 600,000. In (Schep et al., 2021) only barcodes were considered that were represented in at least 50 cells estimated by this approximation.

### 4.3 Downstream Analysis

After quality control and filtering, indel ratios can be calculated. In (Schep et al., 2021) this was done by normalizing each replicate over library size and biological replicates were averaged. The frequency of each indel type as proportion of total reads was calculated on that average. Pathway frequency per IPR was calculated as a proportion of the specific mutation over all indels (excluding wild-type sequences).

It is important to consider that wild-type (wt) sequences can be of various origins during the analysis. They can be either uncut or perfectly repaired by HR, NHEJ or another pathway. Additionally, wt sequences could undergo cycles of cutting and repair as long as they are repaired perfectly. However, we previously estimated that perfect repair of this reporter sequence is rare (Brinkman et al., 2018). As many factors can affect the abundance of the wt sequence in the cells, it is crucial to not overinterpret these results and take cell viability, transfection efficiency and various repair pathway activities into account.

The next step is to classify indels in pathways. Schep et al., 2021 used multiple gRNA target sites within the LBR gene, the main one was termed “LBR2” (“LBR1” in Brinkman et al., 2014) (Brinkman et al., 2014; Brinkman et al., 2018; Schep et al., 2021). This target site was used since it had a predictable repair outcome with very prominent outcomes for MMEJ events (7 bp deletions, flanked by 3 bp microhomologies) and NHEJ events (1 bp insertions). 7 bp deletions were classified as MMEJ, and 1 bp insertions were classified as NHEJ. Schep et al., 2021 focused on the balance between these outcomes (Brinkman et al., 2014; Brinkman et al., 2018; Schep et al., 2021). This classification can easily be adapted for different guides, when clear microhomologies and NHEJ outcomes are known. To discover which indels are caused by the NHEJ pathway, in Schep et al., 2021 the M3814 inhibitor was used (Schep et al., 2021). Indels with an indel ratio of at least 0.01 in the DMSO setting and a significant decrease (adjusted *p*-value < 0.05) in the M3814 inhibitor setting (one-sided Wilcoxon test) were classified as NHEJ. Alternatively, for guides that result in a broader spectrum of multiple mutations, a pipeline setting can be used to detect specific MMEJ events. This alternative pipeline setting performs pairwise alignments with the wild-type sequence was used, resulting in more detailed scar information at the cost of extra computational time. Adapting this strategy for LBR2, other microhomologies can be identified. These include a 14 bp deletion with a 3 bp microhomology and a 22 bp deletion with a 6 bp microhomology.

After mutation counts are separated by MMEJ and NHEJ (and unclassified) events, different calculations can be made for pathway balance and mutational rates. To calculate basic mutational rates a division over total indel can be a simple measure. For comparison in pathway balance between NHEJ and MMEJ, a relative pathway proportion can be calculated using: $\frac{MMEJ}{\left(MMEJ+NHEJ\right)}$ .

## 5 Materials and Methods

### 5.1 Sequences

1. DSB-TRIP plasmids (sequences available at https://osf.io/k2fwt/files/)
  a. EB007
  b. EB011 (EB011_pPTK-P.CMV.584-eGFP-trim1-PI04_mutated)
  c. pPTK-BC-IPR (GenBank: MW408732)
  d. EB032_pBKS-sgRNA
  e. EB032_pBKS-sgRNA-LBR2
2. Oligos

**Table:** 

Name	Number	Sequence (5′ -> 3′)
barcoding-primer-fw	TAC0003	ACTGATCATGGGTACCGATCA**(N)16**TTGTGGCCGGCCCTTGTGACCTGCA
barcoding-primer-rv	TAC0004	AAA​AGC​GCG​CAT​ACT​AGA​TTA​ACC​CTA​GAA​AGA​TAA​TCA​TAT​TG
TIDE_endo_LBR_F	TAC0017	GTA​GCC​TTT​CTG​GCC​CTA​AAA​T
TIDE_endo_LBR_R	TAC0018	AAA​TGG​CTG​TCT​TTC​CCA​GTA​A
indelPCR1-fw-indexed	TAC0007.1–24	ACACTCTTTCCCTACACGACGCTCTTCCGATCT**(N)10**GTCACAAGGGCCGGCCACA
indelPCR1-rv	TAC0012	GTG​ACT​GGA​GTT​CAG​ACG​TGT​GCT​CTT​CCG​ATC​T
indelPCR2-fw	TAC0009	AAT​GAT​ACG​GCG​ACC​ACC​GAG​ATC​TAC​ACT​CTT​TCC​CTA​CAC​GAC​GCT​CTT​CCG​ATC​T
indelPCR2-rv	TAC0011	CAA​GCA​GAA​GAC​GGC​ATA​CGA​GAT​GTG​ACT​GGA​GTT​CAG​ACG​TGT​GCT​CTT​CCG​ATC​T
indelPCR2-rv-indexed	TAC0159.1–96	CAAGCAGAAGACGGCATACGAGAT**(N)6**GTGACTGGAGTTCAGACGTGTGCTCTTCCGATCT
Sanger_map_IPR_F	TAC0065	ATG​CTA​GCG​TGA​CTG​GAG​TT

### 5.2 Reagents

#### 5.2.1 *In Vitro* Applications

1. Nuclease-free water
2. Agarose MP (Roche Cat#: 11388991001; brand not critical)
3. Ethidium bromide (EtBr; Sigma-Aldrich, Cat#: E8751; brand not critical)
4. GeneRuler 100 bp Plus DNA Ladder (ThermoFisher, Cat#: SM0321)
5. 50× TAE (Lonza, cat. no. 51216; brand not critical)
6. MyTac Red Mix 2× (Bioline—Cat#: BIO-25044; brand not critical)
7. NheI-HF^®^ (New England BioLabs—Cat#: R3131S/L—20 U/µl)
8. KpnI-HF^®^ (New England BioLabs—Cat#: R3142S/L—20 U/µl)
9. BssHII (New England BioLabs—Cat#: R0199S/L—5 U/µl)
10. MluI-HF (New England BioLabs—Cat#: R0198S/L—20 U/µl)
11. Ampicilin (brand not critical)
12. LB-Agar (brand not critical)
13. Culture dishes, 10 cm (brand not critical)
14. Lysogeny broth (LB; brand not critical)
15. Shrimp Alkaline Phosphatase (New England BioLabs—Cat#: M0371S—1 U/µl)
16. T4 DNA ligase (Roche Cat#: 10799009001—5 U/µl)
17. Exonuclease I (New England BioLabs—Cat#: M0293S)
18. dNTP Mix (Bioline—Cat#: BIO-39043; brand not critical)
19. Phusion^®^ HF DNA Polymerase (New England BioLabs—Cat#: M0530L)
20. CleanPCR Beads (CleanNA—Cat#: CPCR-0500)

#### 5.2.2 Tissue Culture

1. ddCas9-K562 cell line (our lab)
2. Tissue culture medium, for K562: RPMI 1640 (GIBCO—Cat#: 21875034)
3. Fetal Bovine Serum (Sigma—Cat#: F7524)
4. Penicillin-Streptomycin (5,000 U/ml) (GIBCO–Cat#: 15070063)
5. PBS
6. T-75 tissue culture flasks
7. 10 cm tissue culture dishes
8. 15 ml falcons
9. 50 ml falcons
10. Lipofectamine 2000 (Invitrogen—Cat#: 11668019)
11. Tamoxifen (resuspended in DMSO at 1 mM; Sigma-Aldrich—Cat#: T5648)
12. Dimethyl sulfoxide (DMSO; Sigma—Cat#: D4540)
13. 50 ml seringe
14. Sterile 0.22 µM filter
15. KH_2_PO_4_ (e.g., Sigma-Aldrich—Cat# P5655)
16. NaHCO_3_ (e.g., Sigma-Aldrich—Cat# S5761)
17. MgCl_2_ (e.g., Sigma-Aldrich—Cat# M8266)
18. Glucose (e.g., Sigma-Aldrich—Cat# G7021)
19. ATP 100 mM (Thermo Scientific—Cat#: R0441)
20. Shield-1 (resuspended in 100% Ethanol at 500 µM; Aeobius—Cat#: AOB1848)

#### 5.2.3 Bacterial Strains

1. CloneCatcher DH5α electrocompetent *E. coli* (Genlantis—Cat# C810111)
2. JM109 competent cells (Promega—Cat#: L2001)

#### 5.2.4 Commercial Kits

1. PCR Isolate II PCR and Gel Kit (Bioline, Cat#: BIO-52060; brand not critical)
2. PureLink™ HQ Mini Plasmid DNA Purification Kit (ThermoFisher, Cat#: K210001; brand not critical)
3. PureLink™ HiPure Plasmid Filter Maxiprep Kit (ThermoFisher, Cat#: K210017; brand not critical)
4. Lipofectamine 2000 (Invitrogen, Cat#: 11668019)

#### 5.2.5 Hardware

1. NanoDrop spectrophotometer (ThermoFisher) or equivalent
2. DNA SpeedVac (New Brunswick Scientific) or equivalent
3. PCR thermocycler (Bio-Rad) or equivalent
4. Microfuge centrifuge (Eppendorf) or equivalent
5. Vortex (VWR) or equivalent
6. Thermomixer (Eppendorf) or equivalent
7. Gel apparatus for electrophoresis (Bio-Rad) or equivalent
8. Gene Pulser™ electroporator with the Capacitance Extender and Pulse Controller modules (Bio-Rad) or equivalent
9. Cell incubator, CO2 (5%) (Thermo Scientific) or equivalent
10. TC20 automated cell counter (Bio-Rad) or equivalent
11. Amaxa 2D Nucleofector (Lonza)
12. MoFlo^®^ Astrios™ Cell Sorter equipped with 561 nm laser (Beckman Coulter) or equivalent

#### 5.2.6 Software

NOTE: All computational software except for *inDelphi* and *SelfTarget* comes with the conda environment as described above. A script is provided on the GitHub to download these additional dependencies.

1. Conda v4.10.3
2. Bowtie2 v2.3.4
3. Samtools v1.5
4. Cutadapt v1.9.1
5. Starcode v1.1
6. inDelphi
7. FORECasT
8. R version 3.6.3 (2020-02-29)

## Data Availability

The datasets presented in this study can be found in online repositories. The names of the repository/repositories and accession number(s) can be found below: https://osf.io/k2fwt. Plasmids and cell lines generated in this study are available upon request. Plasmid maps and example data is available at: https://osf.oi/k2fwt/. Code is available at: https://github.com/vansteensellab/DSB_TRIP_protocol.

## References

[B1] Akhtar W. de Jong J. Pindyurin A. V. Pagie L. Meuleman W. de Ridder J. (2013). Chromatin Position Effects Assayed by Thousands of Reporters Integrated in Parallel. Cell 154, 914–927. 10.1016/j.cell.2013.07.018 23953119

[B2] Akhtar W. Pindyurin A. V. de Jong J. Pagie L. Ten Hoeve J. Berns A. (2014). Using TRIP for Genome-wide Position Effect Analysis in Cultured Cells. Nat. Protoc. 9, 1255–1281. 10.1038/nprot.2014.072 24810036

[B3] Allen F. Crepaldi L. Alsinet C. Strong A. J. Kleshchevnikov V. De Angeli P. (2018). Predicting the Mutations Generated by Repair of Cas9-Induced Double-Strand Breaks. Nat. Biotechnol. 37, 64–72. 10.1038/nbt.4317 PMC6949135 30480667

[B4] Anzalone A. V. Koblan L. W. Liu D. R. (2020). Genome Editing with CRISPR-Cas Nucleases, Base Editors, Transposases and Prime Editors. Nat. Biotechnol. 38, 824–844. 10.1038/s41587-020-0561-9 32572269

[B5] Aymard F. Bugler B. Schmidt C. K. Guillou E. Caron P. Briois S. (2014). Transcriptionally Active Chromatin Recruits Homologous Recombination at DNA Double-Strand Breaks. Nat. Struct. Mol. Biol. 21, 366–374. 10.1038/nsmb.2796 24658350 PMC4300393

[B6] Banaszynski L. A. Chen L.-c. Maynard-Smith L. A. Ooi A. G. L. Wandless T. J. (2006). A Rapid, Reversible, and Tunable Method to Regulate Protein Function in Living Cells Using Synthetic Small Molecules. Cell 126, 995–1004. 10.1016/j.cell.2006.07.025 16959577 PMC3290523

[B7] Berkovich E. Monnat R. J. Jr. Kastan M. B. (2007). Roles of ATM and NBS1 in Chromatin Structure Modulation and DNA Double-Strand Break Repair. Nat. Cel Biol 9, 683–690. 10.1038/ncb1599 17486112

[B8] Boch J. Scholze H. Schornack S. Landgraf A. Hahn S. Kay S. (2009). Breaking the Code of DNA Binding Specificity of TAL-type III Effectors. Science 326, 1509–1512. 10.1126/science.1178811 19933107

[B9] Brandsma I. Gent D. C. (2012). Pathway Choice in DNA Double Strand Break Repair: Observations of a Balancing Act. Genome Integr. 3, 9. 10.1186/2041-9414-3-9 23181949 PMC3557175

[B10] Brinkman E. K. Chen T. Amendola M. van Steensel B. (2014). Easy Quantitative Assessment of Genome Editing by Sequence Trace Decomposition. Nucleic Acids Res. 42, e168. 10.1093/nar/gku936 25300484 PMC4267669

[B11] Brinkman E. K. Chen T. de Haas M. Holland H. A. Akhtar W. van Steensel B. (2018). Kinetics and Fidelity of the Repair of Cas9-Induced Double-Strand DNA Breaks. Mol. Cel 70, 801–813. e806. 10.1016/j.molcel.2018.04.016 PMC5993873 29804829

[B12] Brinkman E. K. van Steensel B. (2019). Rapid Quantitative Evaluation of CRISPR Genome Editing by TIDE and TIDER. Methods Mol. Biol. 1961, 29–44. 10.1007/978-1-4939-9170-9_3 30912038

[B13] Ceccaldi R. Rondinelli B. D’Andrea A. D. (2016). Repair Pathway Choices and Consequences at the Double-Strand Break. Trends Cel Biol. 26, 52–64. 10.1016/j.tcb.2015.07.009 PMC4862604 26437586

[B14] Chakrabarti A. M. Henser-Brownhill T. Monserrat J. Poetsch A. R. Luscombe N. M. Scaffidi P. (2019). Target-Specific Precision of CRISPR-Mediated Genome Editing. Mol. Cel 73, 699–713. e696. 10.1016/j.molcel.2018.11.031 PMC6395888 30554945

[B15] Chang H. H. Y. Pannunzio N. R. Adachi N. Lieber M. R. (2017). Non-homologous DNA End Joining and Alternative Pathways to Double-Strand Break Repair. Nat. Rev. Mol. Cel Biol 18, 495–506. 10.1038/nrm.2017.48 PMC7062608 28512351

[B16] Chen W. McKenna A. Schreiber J. Haeussler M. Yin Y. Agarwal V. (2019). Massively Parallel Profiling and Predictive Modeling of the Outcomes of CRISPR/Cas9-mediated Double-Strand Break Repair. Nucleic Acids Res. 47, 7989–8003. 10.1093/nar/gkz487 31165867 PMC6735782

[B17] Cong L. Ran F. A. Cox D. Lin S. Barretto R. Habib N. (2013). Multiplex Genome Engineering Using CRISPR/Cas Systems. Science 339, 819–823. 10.1126/science.1231143 23287718 PMC3795411

[B18] De Sandre-Giovannoli A. Chaouch M. Kozlov S. Vallat J.-M. Tazir M. Kassouri N. (2002). Homozygous Defects in LMNA, Encoding Lamin A/C Nuclear-Envelope Proteins, Cause Autosomal Recessive Axonal Neuropathy in Human (Charcot-Marie-Tooth Disorder Type 2) and Mouse. Am. J. Hum. Genet. 70, 726–736. 10.1086/339274 11799477 PMC384949

[B19] Eriksson M. Brown W. T. Gordon L. B. Glynn M. W. Singer J. Scott L. (2003). Recurrent De Novo point Mutations in Lamin A Cause Hutchinson-Gilford Progeria Syndrome. Nature 423, 293–298. 10.1038/nature01629 12714972 PMC10540076

[B20] Gaj T. Gersbach C. A. Barbas C. F. 3rd (2013). ZFN, TALEN, and CRISPR/Cas-based Methods for Genome Engineering. Trends Biotechnol. 31, 397–405. 10.1016/j.tibtech.2013.04.004 23664777 PMC3694601

[B21] Giannoukos G. Ciulla D. M. Marco E. Abdulkerim H. S. Barrera L. A. Bothmer A. (2018). UDiTaS, a Genome Editing Detection Method for Indels and Genome Rearrangements. BMC Genomics 19, 212. 10.1186/s12864-018-4561-9 29562890 PMC5861650

[B22] Gisler S. Gonçalves J. P. Akhtar W. de Jong J. Pindyurin A. V. Wessels L. F. A. (2019). Multiplexed Cas9 Targeting Reveals Genomic Location Effects and gRNA-Based Staggered Breaks Influencing Mutation Efficiency. Nat. Commun. 10, 1598. 10.1038/s41467-019-09551-w 30962441 PMC6453899

[B23] Goldstein M. Derheimer F. A. Tait-Mulder J. Kastan M. B. (2013). Nucleolin Mediates Nucleosome Disruption Critical for DNA Double-Strand Break Repair. Proc. Natl. Acad. Sci. 110, 16874–16879. 10.1073/pnas.1306160110 24082117 PMC3801049

[B24] Hussmann J. A. Ling J. Ravisankar P. Yan J. Cirincione A. Xu A. (2021). Mapping the Genetic Landscape of DNA Double-Strand Break Repair. Cell 184, 5653–5669. e5625. 10.1016/j.cell.2021.10.002 34672952 PMC9074467

[B25] Iacovoni J. S. Caron P. Lassadi I. Nicolas E. Massip L. Trouche D. (2010). High-resolution Profiling of γH2AX Around DNA Double Strand Breaks in the Mammalian Genome. EMBO J. 29, 1446–1457. 10.1038/emboj.2010.38 20360682 PMC2868577

[B26] Iliakis G. Murmann T. Soni A. (2015). Alternative End-Joining Repair Pathways Are the Ultimate Backup for Abrogated Classical Non-homologous End-Joining and Homologous Recombination Repair: Implications for the Formation of Chromosome Translocations. Mutat. Research/Genetic Toxicol. Environ. Mutagenesis 793, 166–175. 10.1016/j.mrgentox.2015.07.001 26520387

[B27] Jain S. Shukla S. Yang C. Zhang M. Fatma Z. Lingamaneni M. (2021). TALEN Outperforms Cas9 in Editing Heterochromatin Target Sites. Nat. Commun. 12, 606. 10.1038/s41467-020-20672-5 33504770 PMC7840734

[B28] Jinek M. Chylinski K. Fonfara I. Hauer M. Doudna J. A. Charpentier E. (2012). A Programmable Dual-RNA-Guided DNA Endonuclease in Adaptive Bacterial Immunity. Science 337, 816–821. 10.1126/science.1225829 22745249 PMC6286148

[B29] Jinek M. East A. Cheng A. Lin S. Ma E. Doudna J. (2013). RNA-programmed Genome Editing in Human Cells. Elife 2, e00471. 10.7554/eLife.00471 23386978 PMC3557905

[B30] Kallimasioti-Pazi E. M. Thelakkad Chathoth K. Taylor G. C. Meynert A. Ballinger T. Kelder M. J. E. (2018). Heterochromatin Delays CRISPR-Cas9 Mutagenesis but Does Not Influence the Outcome of Mutagenic DNA Repair. Plos Biol. 16, e2005595. 10.1371/journal.pbio.2005595 30540740 PMC6306241

[B31] Kim J. Sturgill D. Tran A. D. Sinclair D. A. Oberdoerffer P. (2016). Controlled DNA Double-Strand Break Induction in Mice Reveals post-damage Transcriptome Stability. Nucleic Acids Res. 44, e64. 10.1093/nar/gkv1482 26687720 PMC4838352

[B32] Langmead B. Salzberg S. L. (2012). Fast Gapped-Read Alignment with Bowtie 2. Nat. Methods 9, 357–359. 10.1038/nmeth.1923 22388286 PMC3322381

[B33] Lemaître C. Grabarz A. Tsouroula K. Andronov L. Furst A. Pankotai T. (2014). Nuclear Position Dictates DNA Repair Pathway Choice. Genes Dev. 28, 2450–2463. 10.1101/gad.248369.114 25366693 PMC4233239

[B34] Lin S. Staahl B. T. Alla R. K. Doudna J. A. (2014). Enhanced Homology-Directed Human Genome Engineering by Controlled Timing of CRISPR/Cas9 Delivery. Elife 3, e04766. 10.7554/eLife.04766 25497837 PMC4383097

[B35] Mali P. Yang L. Esvelt K. M. Aach J. Guell M. DiCarlo J. E. (2013). RNA-guided Human Genome Engineering via Cas9. Science 339, 823–826. 10.1126/science.1232033 23287722 PMC3712628

[B36] Martin M. (2011). Cutadapt Removes Adapter Sequences from High-Throughput Sequencing Reads. EMBnet j. 17, 10. 10.14806/ej.17.1.200

[B37] Massip L. Caron P. Iacovoni J. S. Trouche D. Legube G. (2010). Deciphering the Chromatin Landscape Induced Around DNA Double Strand Breaks. Cell Cycle 9, 3035–3044. 10.4161/cc.9.15.12412 20714222

[B38] McVey M. Lee S. E. (2008). MMEJ Repair of Double-Strand Breaks (Director's Cut): Deleted Sequences and Alternative Endings. Trends Genet. 24, 529–538. 10.1016/j.tig.2008.08.007 18809224 PMC5303623

[B39] Mölder F. Jablonski K. P. Letcher B. Hall M. B. Tomkins-Tinch C. H. Sochat V. (2021). Sustainable Data Analysis with Snakemake. F1000Res 10, 33. 10.12688/f1000research.29032.2 34035898 PMC8114187

[B40] Moscou M. J. Bogdanove A. J. (2009). A Simple Cipher Governs DNA Recognition by TAL Effectors. Science 326, 1501. 10.1126/science.1178817 19933106

[B41] Niu Y. Tenney K. Li H. Gimble F. S. (2008). Engineering Variants of the I-SceI Homing Endonuclease with Strand-specific and Site-specific DNA-Nicking Activity. J. Mol. Biol. 382, 188–202. 10.1016/j.jmb.2008.07.010 18644379 PMC2700736

[B42] Pokusaeva V. O. Diez A. R. Espinar L. Filion G. J. (2021). Strand Asymmetry Influences Mismatch Resolution during Single-Strand Annealing. bioRxiv. 10.1101/847160v2 PMC9001825 35414014

[B43] Richardson C. D. Kazane K. R. Feng S. J. Zelin E. Bray N. L. Schäfer A. J. (2018). CRISPR-Cas9 Genome Editing in Human Cells Occurs via the Fanconi Anemia Pathway. Nat. Genet. 50, 1132–1139. 10.1038/s41588-018-0174-0 30054595

[B44] Richardson C. D. Ray G. J. DeWitt M. A. Curie G. L. Corn J. E. (2016). Enhancing Homology-Directed Genome Editing by Catalytically Active and Inactive CRISPR-Cas9 Using Asymmetric Donor DNA. Nat. Biotechnol. 34, 339–344. 10.1038/nbt.3481 26789497

[B45] Schep R. Brinkman E. K. Leemans C. Vergara X. van der Weide R. H. Morris B. (2021). Impact of Chromatin Context on Cas9-Induced DNA Double-Strand Break Repair Pathway Balance. Mol. Cel 81, 2216–2230. e2210. 10.1016/j.molcel.2021.03.032 PMC8153251 33848455

[B46] Scully R. Panday A. Elango R. Willis N. A. (2019). DNA Double-Strand Break Repair-Pathway Choice in Somatic Mammalian Cells. Nat. Rev. Mol. Cel Biol 20, 698–714. 10.1038/s41580-019-0152-0 PMC7315405 31263220

[B47] Shen M. W. Arbab M. Hsu J. Y. Worstell D. Culbertson S. J. Krabbe O. (2018). Predictable and Precise Template-free CRISPR Editing of Pathogenic Variants. Nature 563, 646–651. 10.1038/s41586-018-0686-x 30405244 PMC6517069

[B48] van Overbeek M. Capurso D. Carter M. M. Thompson M. S. Frias E. Russ C. (2016). DNA Repair Profiling Reveals Nonrandom Outcomes at Cas9-Mediated Breaks. Mol. Cel 63, 633–646. 10.1016/j.molcel.2016.06.037 27499295

[B49] Vítor A. C. Huertas P. Legube G. de Almeida S. F. (2020). Studying DNA Double-Strand Break Repair: An Ever-Growing Toolbox. Front. Mol. Biosci. 7, 24. 10.3389/fmolb.2020.00024 32154266 PMC7047327

[B50] Wienert B. Nguyen D. N. Guenther A. Feng S. J. Locke M. N. Wyman S. K. (2020). Timed Inhibition of CDC7 Increases CRISPR-Cas9 Mediated Templated Repair. Nat. Commun. 11, 2109. 10.1038/s41467-020-15845-1 32355159 PMC7193628

[B51] Zetsche B. Gootenberg J. S. Abudayyeh O. O. Slaymaker I. M. Makarova K. S. Essletzbichler P. (2015). Cpf1 Is a Single RNA-Guided Endonuclease of a Class 2 CRISPR-Cas System. Cell 163, 759–771. 10.1016/j.cell.2015.09.038 26422227 PMC4638220

[B52] Zhou B. Ho S. S. Greer S. U. Zhu X. Bell J. M. Arthur J. G. (2019). Comprehensive, Integrated, and Phased Whole-Genome Analysis of the Primary ENCODE Cell Line K562. Genome Res. 29, 472–484. 10.1101/gr.234948.118 30737237 PMC6396411

[B53] Zorita E. Cuscó P. Filion G. J. (2015). Starcode: Sequence Clustering Based on All-Pairs Search. Bioinformatics 31, 1913–1919. 10.1093/bioinformatics/btv053 25638815 PMC4765884

